# Comparative inhibitory effects of bepotastine and diphenhydramine on rituximab-induced infusion reactions

**DOI:** 10.1007/s12185-025-03990-6

**Published:** 2025-04-29

**Authors:** Tomoki Hori, Kazuhiro Yamamoto, Tomoaki Nakagawa, Rinako Nakagawa, Masami Okayama, Tamika Sudou, Moe Hamasaki, Mai Yasuda, Shinya Kobayashi, Fumihiko Nakamura, Hideo Yagi, Yumi Kitahiro, Shigeki Ikushima, Ikuko Yano

**Affiliations:** 1 Department of Pharmacy, Nara Prefecture General Medical Center, 2-897-5 Shichijo-Nishimachi, Nara, 630-8581 Japan; 2 Department of Pharmacy, Kobe University Hospital, 7-5-2 Kusunoki-Cho, Chuo-Ku, Kobe, 650-0017 Japan; 3 Department of Integrated Clinical and Basic Pharmaceutical Sciences, Faculty of Medicine, Dentistry and Pharmaceutical Sciences, Okayama University, 2-5-1, Shikata-Cho, Kita-Ku, Okayama, 700-8558 Japan; 4 Department of Hematology and Oncology, Nara Prefecture General Medical Center, 2-897-5 Shichijo-Nishimachi, Nara, 630-8581 Japan; 5 Department of Laboratory Medicine, Nara Prefecture General Medical Center, 2-897-5 Shichijo-Nishimachi, Nara, 630-8581 Japan

**Keywords:** Rituximab, Infusion reaction, Bepotastine, Diphenhydramine

## Abstract

**Purpose:**

Infusion-related reaction (IRR) is a common adverse event induced by rituximab. Although first-generation histamine 1 receptor antagonists (H1RAs) are commonly used to prevent IRR, evidence on IRR suppression by the second-generation H1RA bepotastine is scarce. In this study, we assessed the inhibitory effects of bepotastine on rituximab-induced IRR and compared them with those of the first-generation H1RA diphenhydramine.

**Methods:**

We retrospectively evaluated IRR incidence in patients with B-cell non-Hodgkin lymphoma who received their first dose of rituximab.

**Results:**

The incidence of any grade IRR was 9.8% in the bepotastine group (*n* = 92), which was significantly lower than the 30.2% rate in the diphenhydramine group (*n* = 96; *p* < 0.001). The incidence of grade 2 or higher IRR was similar between the two groups (6.5% vs. 12.5%; *p* = 0.16). Multivariable logistic regression analysis revealed that the risk of any grade IRR incidence was higher in patients with B symptoms and bulky disease. Premedication with bepotastine was an independent factor in reducing the risk of any grade IRR incidence (odds ratio = 0.19, 95% confidence interval: 0.08–0.47).

**Conclusion:**

Bepotastine may be more effective than diphenhydramine in reducing the incidence of rituximab-induced IRR, particularly low-grade reactions.

## Introduction

Rituximab, a human/murine, chimeric anti-CD20 monoclonal antibody, has established efficacy and a favorable and well-defined safety profile in patients with CD20-expressing lymphoid malignancies, including indolent and aggressive forms of B-cell non-Hodgkin’s lymphoma (B-NHL) [1]. Although rituximab has a generally acceptable toxicity profile, it can cause infusion-related reactions (IRR), such as fever, chills, nausea, rash, rigor, and hypotension. The spectrum of IRR varies from mild to fatal, with 80% of the fatal IRRs occurring with the first rituximab infusion [2], the most severe of which occur within 30–120 min after the first infusion [3]. Rituximab-induced IRR causes treatment interruptions and delays and often requires the administration of rescue medication, creating additional burdens on patients and medical practitioners. Therefore, pharmacological prophylaxis with antihistamines, corticosteroids, or both is generally recommended to reduce the incidence and severity of IRRs [3]. Furthermore, because rituximab is often included in the regimen for ambulant chemotherapy, using a safer premedication is desirable.

Diphenhydramine or d-chlorpheniramine, which is a first-generation histamine 1 receptor antagonist (H1RA), has been used for the prevention of rituximab-induced IRR in a national phase I clinical trial [4] and a national phase II clinical trial [5]. Thus, many studies have reported the use of first-generation H1RAs as prophylaxis against IRRs [3, 6, 7]. First-generation H1RAs have low receptor selectivity and produce antimuscarinic, anti-α-adrenergic, and antiserotonergic effects. They also readily cross the blood–brain barrier and inhibit histamine-induced neurotransmission at central nervous system (CNS) H1 receptors, causing drowsiness, sedation, somnolence, and fatigue [8]. First-generation H1RAs have been associated with a significantly increased risk of falls and fractures in elderly patients [9].

Bepotastine, a second-generation H1RA, is a selective histamine receptor antagonist indicated for alleviating symptoms of allergic rhinitis, urticaria, and pruritus associated with skin diseases. Bepotastine offers three clinical advantages as a premedication for rituximab-induced IRR. First, second-generation H1RAs cause milder drowsiness than first-generation H1RAs [10]. Secondly, diphenhydramine has an anticholinergic effect and is contraindicated in Japan for patients with angle-closure glaucoma and benign prostatic hyperplasia, whereas bepotastine has no restrictions on its administration to these patients. Therefore, bepotastine is suited for patients with these complications. Third, according to the package insert of bepotastine, it presents no potential interactions with drugs. However, evidence of the inhibitory effect of bepotastine on IRR is lacking.

This study evaluated the inhibitory effect of premedication with diphenhydramine or bepotastine on rituximab-induced IRR in patients with B-NHL receiving a first dose of rituximab.

## Materials and methods

### Patients

This was a single-center, retrospective observational study conducted using electronic medical records from the Nara Prefecture General Medical Center, Nara, Japan. Considering the clinical advantages of the second-generation H1RA, we changed the H1RA used for premedication in July 2021, from diphenhydramine 50 mg to bepotastine orally disintegrating tablets 10 mg. The inclusion criterion was patients with pathologically confirmed B-NHL who received their first rituximab dose between January 2020 and April 2023 at the Nara Prefecture General Medical Center, Nara, Japan. The exclusion criteria were patients with missing data on the patient characteristics listed in Table 1 and patients taking regular H1RA on the day of rituximab administration. The study population was divided into two groups based on whether they received diphenhydramine or bepotastine as premedication for IRR. Patients administered with steroids before rituximab infusion were also included. Prior administration of steroids was defined as administration of regimen-containing steroids (e.g., day 1 prednisolone administration before day 2 rituximab infusion in the CHOP-R regimen) or other regular oral or intravenous administration of steroids before the day of rituximab infusion. Prior cytotoxic chemotherapy was defined as the administration of a cytotoxic anticancer drug before the date of rituximab infusion. The rituximab dose intensity was calculated by dividing the actual dose by the planned dose. Informed consent was obtained by allowing each patient to opt-out at any time after reviewing the study summary published on the website.

**Table 1:** Characteristics of patients treated with histamine 1 receptor antagonists

Parameters		Diphenhydramine (*n* = 96)	Bepotastine (*n* = 92)	*p*-value
Age (years)	Median (range)	75 (20–93)	75 (45–92)	0.49^b^
Sex	Female, *n* (%)	43 (44.8)	35 (38.0)	0.35^a^
BSA (m^2^)	Median (range)	1.6 (1.2–2.0)	1.6 (1.2–2.0)	0.80^b^
Histological subtypes	Indolent lymphomas, *n* (%)	22 (22.9)	23 (25.0)	0.74^a^
	Aggressive lymphomas, *n* (%)	74 (77.1)	69 (75.0)	
ECOG PS	≥ 2, *n* (%)	5 (5.2)	11 (12.0)	0.10^a^
Stage (Ann Arbor/Lugano)	III, IV, *n* (%)	38 (39.6)	55 (59.8)	0.01^c^
	I, II, *n* (%)	46 (47.9)	27 (29.3)	
	Unclassified, *n* (%)	12 (12.5)	10 (10.9)	
Presence of B symptom	Yes, *n* (%)	18 (18.8)	18 (19.6)	0.89^a^
Presence of bulky disease (≥ 10 cm)	Yes, *n* (%)	7 (7.3)	7 (7.6)	0.93^a^
Presence of bone marrow involvement	Yes, *n* (%)	6 (6.3)	8 (8.7)	0.52^a^
Hb (g/dL)	Median (range)	12.2 (5.7–17.0)	12.1 (7.1–15.5)	0.13^b^
LDH (U/L)	Median (range)	228 (106–28,628)	246 (89–2,316)	0.35^b^
sIL-2R (U/mL)	Median (range)	1,002 (204–27,737)	1,226 (182–17,934)	0.48^b^
Name of regimen	R-CHOP (including R-CVP), *n *(%)	46 (47.9)	49 (53.3)	
	R monotherapy, *n* (%)	45 (46.9)	19 (20.7)	
	Pola-R-CHP, *n* (%)	0 (0)	15 (16.3)	
	Other, *n* (%)	5 (5.2)	9 (9.8)	
Prior administration of steroids before rituximab infusion	Yes, *n* (%)	68 (70.8)	72 (78.3)	0.24^a^
Prior cytotoxic chemotherapy	Yes, *n* (%)	75 (78.1)	72 (78.3)	0.98^a^
Concomitant medication	NSAIDs, Acetaminophen, *n* (%)	15 (15.6)	20 (21.7)	0.28^a^
Rituximab dose intensity (%)	Median (range)	98 (87–100)	99 (88–100)	0.002^b^
Rituximab biosimilar	Yes, *n* (%)	87 (90.6)	91 (98.9)	0.01^a^

All *p* values were calculated using the ^a^ chi-square, ^b^ Mann–Whitney U, or ^c^ chi-square test performed except for unclassified cases

*BSA* body surface area; *ECOG PS* performance status defined by The Eastern Cooperative Oncology Group; *Hb* hemoglobin; *IRR* infusion-related reaction; *LDH* lactate dehydrogenase; *NSAIDs* nonsteroidal anti-inflammatory drugs; *sIL-2R* soluble interleukin-2 receptor

### Rituximab administration

All patients received oral acetaminophen 400 mg and H1RA (diphenhydramine tablet 50 mg or bepotastine tablet 10 mg) approximately 30 min before rituximab administration. Intravenous steroids (hydrocortisone 100 mg) were administered immediately before rituximab administration. Rituximab was diluted with saline solution to a concentration of 1 mg/mL and administered intravenously to patients at a dose of 375 mg/m^2^. Rituximab was initially administered at a rate of 50 mg/h. If no IRR occurred, the dose was increased by 50 mg/h at 30-min intervals up to a dose of 400 mg/h. Patients’ vital signs, including pulse, oxygen saturation, respiratory rate, and body temperature, were carefully monitored during rituximab administration.

### Endpoint

The primary endpoint was the incidence rate of IRR of any grade. IRR to rituximab was defined as the onset of the following symptoms within 24 h of rituximab administration based on the medical records: allergic reaction/sensitivity (including drug fever), arthralgia, bronchospasm, cough, dizziness, dyspnea, fatigue (asthenia, lethargy, malaise), headache, hypertension, hypotension, myalgia, nausea/vomiting, pruritus/itching, rash/scaling, chilling, sweating, tachycardia, tumor pain (new or worsening of tumor pain induced by treatment), urticaria (welts, wheals), angioedema, hypoxia (saturation of percutaneous oxygen [SpO_2_] less than 90%), pulmonary infiltration, acute respiratory distress syndrome, myocardial infarction, ventricular fibrillation, cardiogenic shock, anaphylactoid event, and death [2, 11, 12]. The period from the onset of any grade IRR to the documented time for the resolution of symptoms was calculated. Each IRR was graded according to the Common Terminology Criteria for Adverse Events version 5.0.

### Statistical analysis

Categorical variables were compared using the chi-square test, while continuous variables were compared using the Mann–Whitney *U* test. Logistic regression tests were performed to determine the independent factors associated with the incidence of IRR. In addition to the type of the H1RAs and factors with *p* < 0.05 in the univariable analysis, B symptoms, bulky disease (≥ 10 cm in diameter), and bone marrow involvement, well-known risk factors for IRR [7, 13–15], were added as explanatory variables in the multivariable analysis. Differences in median IRR onset time after rituximab treatment and the period from onset of IRR to symptom resolution between each treatment group were assessed using the log-rank test. *P*-values < 0.05 were considered statistically significant. Statistical analyses were performed using IBM SPSS Statistics version 28.0 (IBM, Armonk, NY, USA).

## Results

### Patient characteristics

In total, 211 patients with various types of B-NHL treated with their first dose of rituximab were enrolled in this study. Patients missing data on pathological classification (*n* = 1) and soluble interleukin-2 receptor (sIL-2R; *n* = 6), and patients taking H1RA regularly (*n* = 16) were excluded. Finally, 188 patients were included in the analysis, 96 who received diphenhydramine premedication and 92 who received bepotastine. Table 1 shows the patients’ characteristics. Histologically, 76.1% of the patients had aggressive lymphoma. The proportions of patients in stages III and IV, rituximab dose intensity, and on rituximab formulation (biosimilar) were significantly higher in the bepotastine group than in the diphenhydramine group. No significant differences in other patient characteristics were observed between the two groups. The regimens combined with rituximab consisted of CHOP (cyclophosphamide, doxorubicin, vincristine, and prednisolone)-based chemotherapy; *n* = 95), Pola-CHP (polatuzumab vedotin, cyclophosphamide, doxorubicin, and prednisolone; *n* = 15), others (*n* = 14), and rituximab monotherapy (*n* = 64). All patients received acetaminophen 400 mg and hydrocortisone 100 mg as IRR prophylaxis other than H1RAs. In total, 140 (74.5%) patients received steroids before rituximab infusion. Furthermore, 147 patients (78.2%) received rituximab after cytotoxic chemotherapy on the first cycle of immunochemotherapy.

### Incidence of IRR with rituximab

**Fig. 1:**
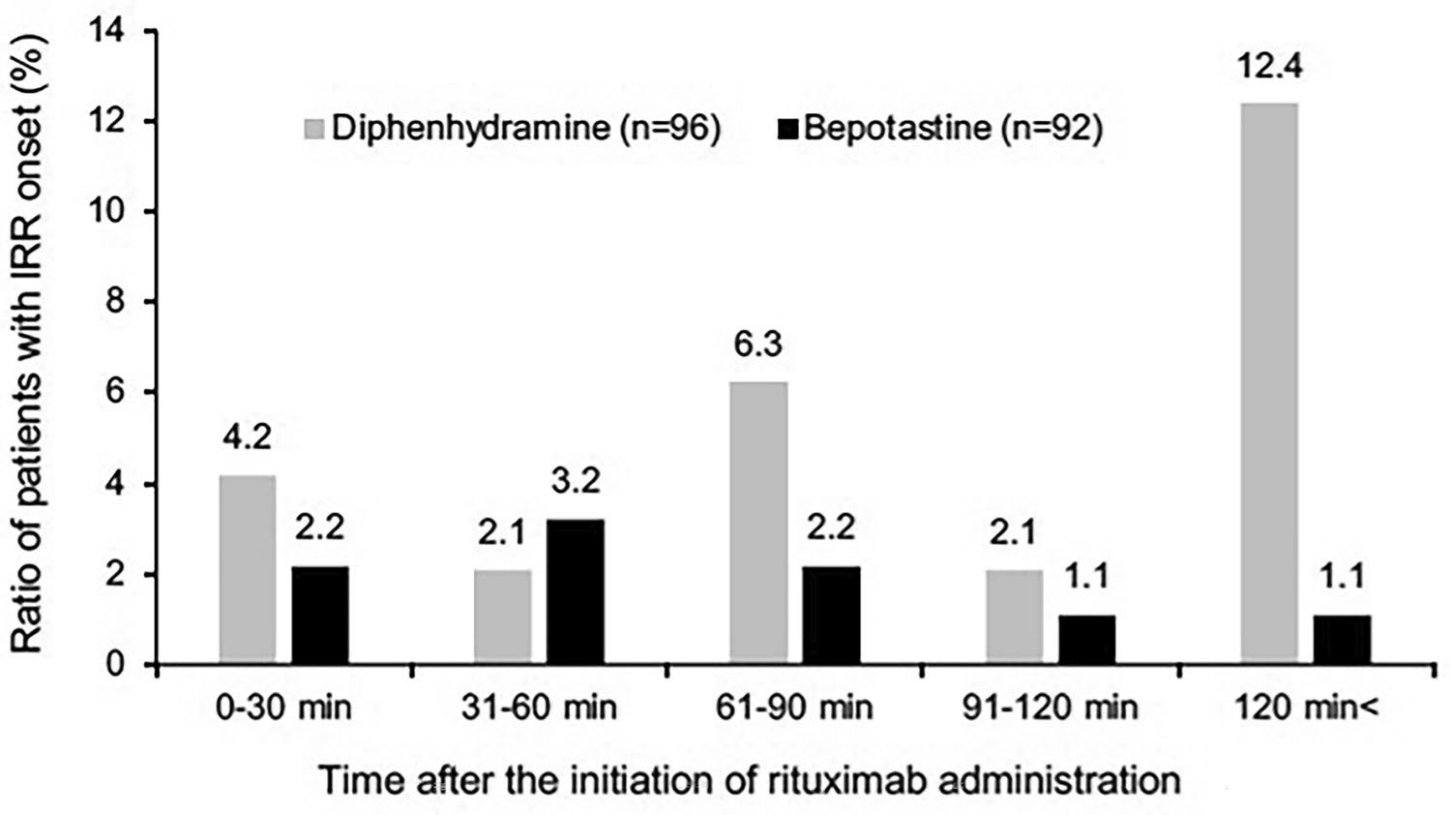
Patient distribution of IRR by time of onset after rituximab administration. Patient distribution is shown except for three patients whose exact onset times of IRR was unknown in the diphenhydramine group. *IRR* infusion-related reaction, diphenhydramine group (*n* = 96), bepotastine group (*n* = 92)

Thirty-eight (20.2%) of the 188 patients experienced any grade of IRR. The incidence of any grade of IRR in the bepotastine group was 9.8%, which was significantly lower than that in the diphenhydramine group at 30.2% (*p* < 0.001; Table 2). The incidence of ≥ grade 2 IRR in the bepotastine group was 6.5%, which did not differ significantly from that of diphenhydramine (12.5%; *p* = 0.16). The median time (min–max) from the initiation of rituximab administration to the onset of IRR was 60 min (30–480 min) in the bepotastine group, which was significantly shorter than that in the diphenhydramine group at 120 min (30–1140 min; *p* = 0.002). The patient distribution by time of IRR onset after the initiation of rituximab is shown in Fig. 1. The distribution of the IRR onset time was highest in the 31–60 min period in the bepotastine group and after 120 min in the diphenhydramine group. The rituximab infusion rate at the onset of IRR, which was after rituximab administration, was evenly distributed from 50 mg/h to 300 mg/h in the diphenhydramine group. Conversely, the rituximab infusion rate at the onset of IRR was distributed between 50 mg/h and 150 mg/h in most patients in the bepotastine group. The distribution of IRR by symptoms was as follows: 10 patients developed an allergic reaction/sensitivity (including drug fever) and experienced nausea/vomiting; 8 patients had dyspnea; and 7 patients developed a rash/scaling and chilling. Among patients who developed any grade IRR (*n* = 38), the period from IRR onset to symptom resolution was unknown for 17 patients due to a lack of information in the electronic medical records. For the remaining 21 patients, the median period (range) from onset of any grade IRR to resolution of symptoms was 30 min (5–150 min) in the diphenhydramine group (*n* = 13) and 60 min (2–253 min) in the bepotastine group (*n* = 8), with no significant difference (*p* = 0.17). Moreover, 17.2% (5/29) of the diphenhydramine group and 22.2% (2/9) of the bepotastine group experienced a relapse of symptoms the day after the first rituximab administration. In addition, 17.2% (5/29) of patients in the diphenhydramine group experienced recurrence of IRR at the time of the second rituximab administration, whereas no patients in the bepotastine group experienced recurrence.

**Table 2:** Incidence and details of infusion-related reactions

		Diphenhydramine (*n* = 96)	Bepotastine (*n* = 92)	*p* -value
Incidence of any grade IRR, *n* (%)		29 (30.2)	9 (9.8)	< 0.001^a^
Incidence of ≥ grade 2 IRR, *n* (%)		12 (12.5)	6 (6.5)	0.16^a^
Time to any grade IRR onset (min)	Median (range)	120 (30–1,140)	60 (30–480)	0.002 ^b^
Infusion rate at the onset of IRR, *n* (%)	50 mg/h	2 (2.1)	1 (1.1)	
	100 mg/h	6 (6.3)	3 (3.3)	
	150 mg/h	5 (5.2)	4 (4.3)	
	200 mg/h	1 (1.0)	0 (0)	
	250 mg/h	3 (3.1)	0 (0)	
	300 mg/h	3 (3.1)	0 (0)	
	After administration	6 (6.3)	1 (1.1)	
	Unknown	3 (3.1)	0 (0)	
Symptoms of IRR, *n* (%)	Allergic reaction/sensitivity (including drug fever)	7 (7.3)	3 (3.3)	
	Nausea/vomiting	6 (6.3)	4 (4.3)	
	Rash/scaling	6 (6.3)	1 (1.1)	
	Tumor pain (new or worsening of tumor pain because of treatment)	5 (5.2)	1 (1.1)	
	Dyspnea	4 (4.2)	4 (4.3)	
	Chilling	4 (4.2)	3 (3.3)	
	Pruritus/itching	3 (3.1)	0 (0)	
	Hypoxia	2 (2.1)	0 (0)	
	Sweating	1 (1.0)	1 (1.1)	
	Headache	1 (1.0)	1 (1.1)	
	Tachycardia	1 (1.0)	0 (0)	
	Arthralgia	1 (1.0)	0 (0)	
	Hypertension	1 (1.0)	0 (0)	
	Fatigue	0 (0)	1 (1.1)	

All *p* values were calculated using the ^a^chi-square or ^b^ log-rank test performed except for three patients in the diphenhydramine group whose exact onset time was unknown. Multiple counts regarding symptoms of IRR were allowed for the same patient. *IRR* infusion-related reaction

### Risk factors for any grade IRR

In the multivariable analysis, B symptoms (odds ratio [OR] = 2.96, 95% confidence interval [CI] 1.20–7.27; *p* = 0.02) and bulky disease (OR = 9.12, 95% CI 2.53–32.9, *p* < 0.001) were significantly associated with the incidence of any grade IRR. Bepotastine use was identified as a significant suppressive factor against the incidence of any grade IRR (OR = 0.19, 95% CI 0.08–0.47; *p* < 0.001) (Table 3).

**Table 3:** Logistic regression analysis of the risk factors of any grade of infusion-related reaction

	Univariable analysis			Multivariable analysis		
Parameters	Odds ratio	95% CI	*p-*value	Odds ratio	95% CI	*p-*value
Histological subtypes (Aggressive)	0.85	0.38–1.93	0.70			
Stage (III, IV)	0.81	0.47–1.41	0.46			
B symptom (Yes)	2.42	1.08–5.46	0.03	2.96	1.20–7.27	0.02
Bone marrow involvement (Yes)	2.37	0.75–7.55	0.14	3.63	0.94–14.0	0.06
Bulky disease (Yes)	6.40	2.07–19.8	0.001	9.12	2.53–32.9	< 0.001
H1RA (bepotastine)	0.25	0.11–0.57	< 0.001	0.19	0.08–0.47	< 0.001
Prior administration of steroids before rituximab infusion (Yes)	0.59	0.27–1.26	0.17			
Prior cytotoxic chemotherapy (Yes)	0.95	0.40–2.26	0.90			

*CI* confidence interval, *H1RA* histamine 1 receptor antagonist

## Discussion

To the best of our knowledge, this is the first retrospective real-world evidence demonstrating that bepotastine, a second-generation H1RA, significantly reduces IRRs induced by rituximab compared with diphenhydramine, as a first-generation H1RA.

In our study, any grade IRR incidence in the bepotastine group was significantly lower than that in the diphenhydramine group. IRR incidence rates may differ between first- and second-generation H1RAs, as well as between second-generation H1RAs [16, 17]. A possible reason for the significant suppression of IRR by bepotastine may be related to the notably lower IRR incidence after 120 min (Fig. 1). Several mechanisms of IRRs induced by rituximab have been reported: immunoglobulin E (IgE)-mediated hypersensitivity, anaphylactoid reaction (IgE-independent allergic reaction), and cytokine release syndrome (CRS) [18]. Additionally, type I hypersensitivity may be another possible CRS mechanism underlying the induction of IRRs by rituximab [19]. Symptom onset of type I hypersensitivity involving IgE is bimodal, with the immediate reaction occurring 30–60 min after antigen exposure and the late-phase reaction, which primarily involves eosinophils, occurring 4–6 h later [20, 21]. The functional mechanism of diphenhydramine is only histamine H1 receptor antagonism. However, bepotastine has an additional antiallergic effect, such as stabilization of mast cell function, inhibition of eosinophilic infiltration, and suppression of interleukin (IL)−5 production, which causes eosinophil migration into the peripheral blood [22]. A possible mechanism of bepotastine suppression of IRRs after 120 min may involve the suppression of eosinophil infiltration, which contributes to the delayed response to rituximab that can occur in type I allergic mechanisms.

Pharmacokinetic properties of diphenhydramine and bepotastine differ in the time to maximum concentration (T_max_). Severe IRR can occur 0.5–2 h after the initiation of rituximab [3]. Tumor necrosis factor-α (TNF-α) and IL-6 are closely involved in the mechanism of IRR development [18]. A previous study found that the T_max_ of TNF-α and IL-6 levels was 1.5 h after rituximab administration [23]. Since the onset of the pharmacological effects of H1RA also depends on T_max_ [24], H1RA with T_max_ closer to 1.5 h is possibly more effective in preventing IRR. The T_max_ of bepotastine besilate tablet 10 mg is 1.0 h [25], whereas the T_max_ of diphenhydramine 50 mg is 1.5 h [26]. Considering that H1RA is administered 0.5 h before rituximab administration, the peak plasma diphenhydramine concentration (approximately 1.0 h after rituximab initiation) should be slightly earlier than with the peak of cytokine release (approximately 1.5 h after rituximab initiation), thereby achieving greater efficacy than bepotastine. However, IRRs that developed more than 2 h after the initiation of rituximab were not adequately suppressed by diphenhydramine (Fig. 1). The elimination half-life (T_1/2_) of bepotastine besilate and diphenhydramine among elderly adults are 2.7 h [25] and 13.5 h [27], respectively. Although diphenhydramine blood concentrations were stable 2 h after rituximab initiation, it does not sufficiently suppress the IRR. Therefore, the antiallergic effect of bepotastine, but not of diphenhydramine, will be responsible for IRR suppression in the later phase. However, further detailed studies are needed to confirm this.

The IRR incidence rate of chlorpheniramine maleate after the first administration of rituximab is 32.9% [13]. In the present study, the IRR incidence in the diphenhydramine group was 30.2%, indicating that the IRR incidence of first-generation H1RAs is comparable to that in the previous report. Furthermore, the incidence of IRR in the bepotastine group was 9.8%, which was comparable to 7.2% for cetirizine [16], another second-generation H1RA. In addition, the findings on IRR symptoms in the present study were similar to those reported by Cho et al. [7].

In this study, the effects of clinical factors, including histological subtypes, cancer stage, B symptoms, bone marrow involvement, bulky disease, and administration of H1RA and steroids before rituximab infusion on IRR incidence was assessed using logistic regression analysis. Rituximab dose intensity and biosimilars were not considered as explanatory variables for the following reasons: for rituximab dose intensity, no differences in influence on IRR incidence were found, and biosimilars are not a risk factor for IRR [28]. In the multivariable analysis, in addition to H1RA administration, B symptoms, bone marrow involvement, and bulky disease, which were identified as independent risk factors for IRRs in previous reports [7, 13–15] were included as explanatory variables. In addition to the clinical markers previously reported as a risk factor for IRR, bepotastine use was identified as an independent factor that reduced IRR.

Our study has some limitations. First, diphenhydramine and bepotastine were used as premedication for rituximab at different times in the study hospital. Due to the retrospective nature of this study, this was unavoidably a comparison of IRRs at different time points. Second, the assessment of IRR was highly subjective, and the accuracy of the information depended on whether patients reported symptoms. Third, the involvement of several physicians in the diagnosis of IRR may lead to diagnostic bias. Fourth, blood concentrations of H1RAs were not measured. Nevertheless, although this is a single-center retrospective study, the post hoc test achieved a detection rate of 93.5%, which is sufficient to guarantee the detection power. Furthermore, a phase II, double-blind, multicenter, randomized trial is currently evaluating the efficacy of first-generation (hydroxyzine pamoate) and second-generation (bepotastine besilate) H1RAs in preventing IRR following initial infusion of rituximab in patients diagnosed with NHL [29]. This prospective study would validate our results [29].

In conclusion, bepotastine may be more effective than diphenhydramine in reducing the incidence of rituximab-induced IRR, particularly low-grade delayed-type reactions.

## Data Availability

The datasets generated during and/or analyzed during the current study are available from the first author upon reasonable request.
